# Immune-related diagnostic markers for benign prostatic hyperplasia and their potential as drug targets

**DOI:** 10.3389/fimmu.2024.1516362

**Published:** 2024-12-05

**Authors:** YaXuan Wang, Jing Wang, Jibin Liu, HaiXia Zhu

**Affiliations:** ^1^ Cancer Research Centre Nantong, Affiliated Tumor Hospital of Nantong University & Nantong Tumor Hospital, Nantong, China; ^2^ Department of Urology, The First Affiliated Hospital of Harbin Medical University, Harbin, China; ^3^ Department of Oncology, Jinling Hospital, Affiliated Hospital of Medical School, Nanjing University, Nanjing, Jiangsu, China

**Keywords:** BPH, biomarkers, machine learning, drug target, immune signatures

## Abstract

**Background:**

Benign prostatic hyperplasia (BPH) is a common issue among older men. Diagnosis of BPH currently relies on imaging tests and assessment of urinary flow rate due to the absence of definitive diagnostic markers. Developing more accurate markers is crucial to improve BPH diagnosis.

**Method:**

The BPH dataset utilized in this study was sourced from the Gene Expression Omnibus (GEO). Initially, differential expression and functional analyses were conducted, followed by the application of multiple machine learning techniques to identify key diagnostic markers. Subsequent investigations have focused on elucidating the functions and mechanisms associated with these markers. The ssGSEA method was employed to evaluate immune cell scores in BPH samples, facilitating the exploration of the relationship between key diagnostic markers and immune cells. Additionally, molecular docking was performed to assess the binding affinity of these key markers to therapeutic drugs for BPH. Tissue samples from BPH patients were collected for experimental validation of the expression differences of the aforementioned genes.

**Result:**

A total of 185 differential genes were identified, comprising 67 up-regulated and 118 down-regulated genes. These genes are implicated in pathways that regulate extracellular matrix tissue composition and cellular responses to transforming growth factor beta stimulation, as well as critical signaling pathways such as AMPK and mTOR. Through the application of various machine learning techniques, DACH1, CACNA1D, STARD13, and RUNDC3B were identified as key diagnostic markers. The ssGSEA algorithm further corroborated the association of these diagnostic genes with diverse immune cells. Moreover, molecular docking analysis revealed strong binding affinities of these markers to tamsulosin and finasteride, suggesting their potential as drug targets. Finally, experimental validation confirmed the expression differences of DACH1, CACNA1D, STARD13, and RUNDC3B in BPH tissues.

**Conclusion:**

This study introduces novel immune-related diagnostic markers for BPH and highlights their promise as new drug targets, providing a valuable approach for predictive diagnosis and targeted therapy of BPH.

## 1 Introduction

Benign prostatic hyperplasia (BPH) is a prevalent disease in middle-aged and older men. Epidemiological studies indicate that the prevalence of BPH increases with age, with rates of 13% between 40-50 years old, 20% among 50-60 years old, 50% between 60-70 years old, 57.1% between 70-80 years old, and 57.1% among 80-90 years old. The prevalence of BPH reaches 83.3% in individuals over 90 years old (1). At present, diagnosing BPH primarily relies on imaging techniques and assessments of urinary flow rates (2). The treatment strategies for BPH mainly include pharmacological therapy and surgical procedures. Both α-blockers and 5α-reductase inhibitors have been proven effective in clinical settings, although their long-term use can result in side effects such as sexual dysfunction, dizziness, headaches, and fatigue (3). Therefore, there is an urgent need for new BPH -specific markers to enable clinicians to diagnose BPH at an earlier stage.

The development of The Cancer Genome Atlas (TCGA) database and the Gene Expression Omnibus (GEO) database has facilitated bioinformatics analysis (4, 5). Weighted gene co-expression network analysis (WGCNA) is a systems biology approach that explores network connections and molecular mechanisms. It is commonly used to analyze large gene expression datasets, identify co-expressed gene clusters, and investigate relationships between gene clusters and sample attributes (6, 7). Machine learning is a field of artificial intelligence that combines statistics and computer science to develop predictive algorithms capable of learning from gene expression datasets to identify key genes (8, 9). Random Forest (RF) is a machine learning algorithm that can effectively classify samples and pinpoint key components that differentiate various groups (10, 11). Despite these advancements, there is a lack of studies that integrate WGCNA and RF methods to discover novel diagnostic markers in BPH samples.

Recent evidence suggests that inflammatory and immune mechanisms are key players in the development of BPH (12, 13). Prostatic inflammation is believed to heighten the risk of progressive BPH by exacerbating LUTS and increasing the likelihood of acute urinary retention (14). Chronic inflammation has been associated with the expansion of prostate tissue, resulting in fibrosis marked by the accumulation of reactive matrix and extracellular matrix (ECM).This matrix deposition can reduce urethral compliance, ultimately causing LUTS (15). Understanding the infiltration of various types of immune cells can aid in the development of novel therapeutic strategies aimed at alleviating symptoms or controlling disease progression through the targeted modulation of specific immune cells or their associated pathways (16). While both innate and adaptive immunity contribute to the development of BPH, the specific molecular mechanisms by which different immune cells impact this condition remain incompletely understood. Therefore, investigating the role of immune cell infiltration in BPH is essential for gaining insights into its molecular pathogenesis from an immunological standpoint. This study aims to identify specific diagnostic markers for BPH using multiple machine learning algorithms and propose new solutions for clinical practice. Additionally, we investigate the correlation between biomarkers and immune cells using omics data.

## 2 Materials and methods

### 2.1 Samples collection

All datasets analyzed in this study were sourced from the GEO database and the downloaded data was in MINiML format. The study included 29 samples of BPH and 3 samples of normal prostate from GSE65343, GSE104749, and GSE119195. For further research, we collected 5 cases each of BPH tissue and normal prostate tissue from Nantong tumor hospital.

### 2.2 Analysis of differences in BPH related genes

BPH samples were classified into BPH and Normal prostate groups. Differential expression of mRNAs was investigated using the Limma package in R software (version 3.40.2.). We defined “p < 0.05, and log2 (fold change) > 1.3 or log2 (fold change) < -1.3” as the threshold mRNA differential expression screening. PCA plots were drawn using the R software package ggord; expression heat maps were displayed using the R software package pheatmap.

### 2.3 Construction of regulatory network of key genes for BPH and analysis of E3 ligase correlation

Interaction network of key genes for BPH and regulatory network of transcription factors analyzed by Gendoma web server (https://ai.citexs.com). The UbiBrowser (http://ubibrowser.bio-it.cn/ubibrowser/) is utilized for the analysis of both predicted and established human networks involving ubiquitin ligase (E3) and its substrates (17). Through the use of UbiBrowser, we conducted an examination of essential E3 ubiquitin ligases which could potentially be influenced by key BPH genes.

### 2.4 Functional analysis of BPH related genes

The Gene Ontology (GO) acts as a widely used tool for gene annotation, placing particular emphasis on molecular functions (MF), biological pathways (BP), and cellular components (CC). An important method for evaluating gene functions and associated high-level genomic functional information is the use of Kyoto Encyclopedia of Genes and Genomes (KEGG) enrichment analysis. In order to gain a deeper understanding of the oncogenic effects of specific genes, we utilized the ClusterProfiler package in R to analyze the GO functions of selected mRNAs and enrich KEGG pathways. For Gene Set Enrichment Analysis (GSEA), we utilized version 3.0 of the GSEA software (18).

### 2.5 Evaluation and correlation analysis of immune infiltration in BPH

Employing the ssGSEA algorithm from the R package GSVA, the analysis of immune cell infiltration in BPH samples was carried out using markers for 28 different immune cell types, as detailed in the publication from Cell Reports (19, 20). Immune infiltration correlation heatmaps were visualized by the R package ggstatsplot. Spearman correlation analysis describes the correlation between quantitative variables that are not normally distributed. After grouping the key genes for BPH in terms of high and low expression, the data were counted accordingly to obtain the percentage of each subgroup in each classification. Stacked bar charts were plotted for the statistics using the ggplot2 package.

### 2.6 Molecular docking of key BPH genes with tamsulosin and finasteride

In order to assess the binding affinity of key genes for BPH and BPH drugs, we employed molecular docking methods for analysis. The CB-Dock2 website served as a useful tool for our investigation, with the Vina score utilized to evaluate the binding affinity of genes to drugs (21). It is commonly accepted that a score <-5.0 kcal/mol indicates a stronger binding interaction between the two entities.

### 2.7 Quantitative real-time polymerase chain reaction (qRT-PCR)

Quantitative real-time polymerase chain reaction (qRT-PCR) was conducted following the extraction of total RNA from prostate tissue using TRIzol reagent (Thermo Fisher, USA). The synthesis of complementary DNA (cDNA) was performed with 500 ng of RNA using HiScript II SuperMix (Vazyme, China). qRT-PCR was executed on an ABI 7500 system (Thermo Fisher, USA) utilizing SYBR Green Master Mix. The PCR amplification conditions comprised 45 cycles, initiated with a 10-minute incubation at 94°C, followed by 10 seconds at 94°C and 45 seconds at 60°C. GAPDH was employed as an internal control. The primer sequence for the target gene is as follows:

### 2.8 Statistical analyses

The statistical differences between two groups were evaluated using a T-test. Spearman method was used to conduct correlation analysis. Any p value below 0.05 was deemed to be statistically significant.

## 3 Results

### 3.1 Analysis of differences based on samples with BPH

**Figure 1:**
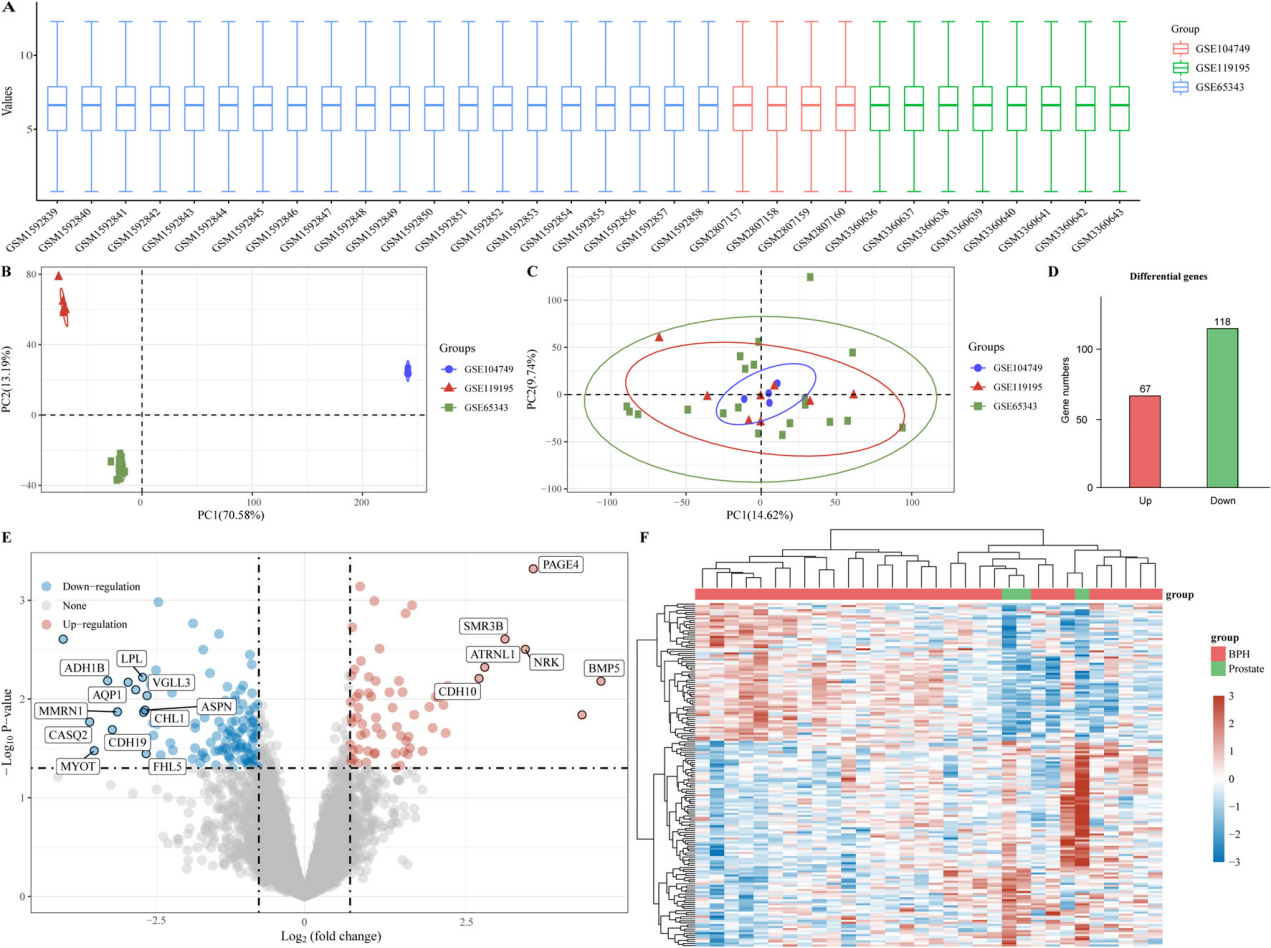
Identification of differential genes for BPH. **(A)** Box plot of standardized data. **(B)** Batch delete previous PCA results for multiple datasets. **(C)** PCA results after batch removal. **(D)** BPH -associated differential genes, with red representing up-regulated genes and green representing down-regulated genes. **(E, F)** Volcano and heat maps for difference analysis.

In order to investigate potential biomarkers for BPH development, we gathered three BPH-related datasets (GSE65343, GSE104749, and GSE119195) from the GEO database, consisting of 29 BPH samples and 3 normal prostate samples. Initially, we normalized and merged the high-throughput sequencing data into a unified dataset for analysis (**Figure 1A**). Subsequently, PCA results were obtained both pre- (**Figure 1B**) and post-batch removal (**Figure 1C**), indicating successful dataset integration for further analysis. Differential analysis comparing the BPH and normal prostate groups was performed using the limma software package in R. Differential gene screening criteria were set at P < 0.05 and log2 (fold change) > 1.3 or log2 (fold change) < -1.3, resulting in the identification of 185 differential genes, including 67 up-regulated genes and 118 down-regulated genes (**Figure 1D**). Moreover, volcano plots and heat maps illustrating the results of the differential analysis were generated (**Figures 1E, F**).

### 3.2 KEGG and GO analysis of BPH-related differential genes

**Figure 2:**
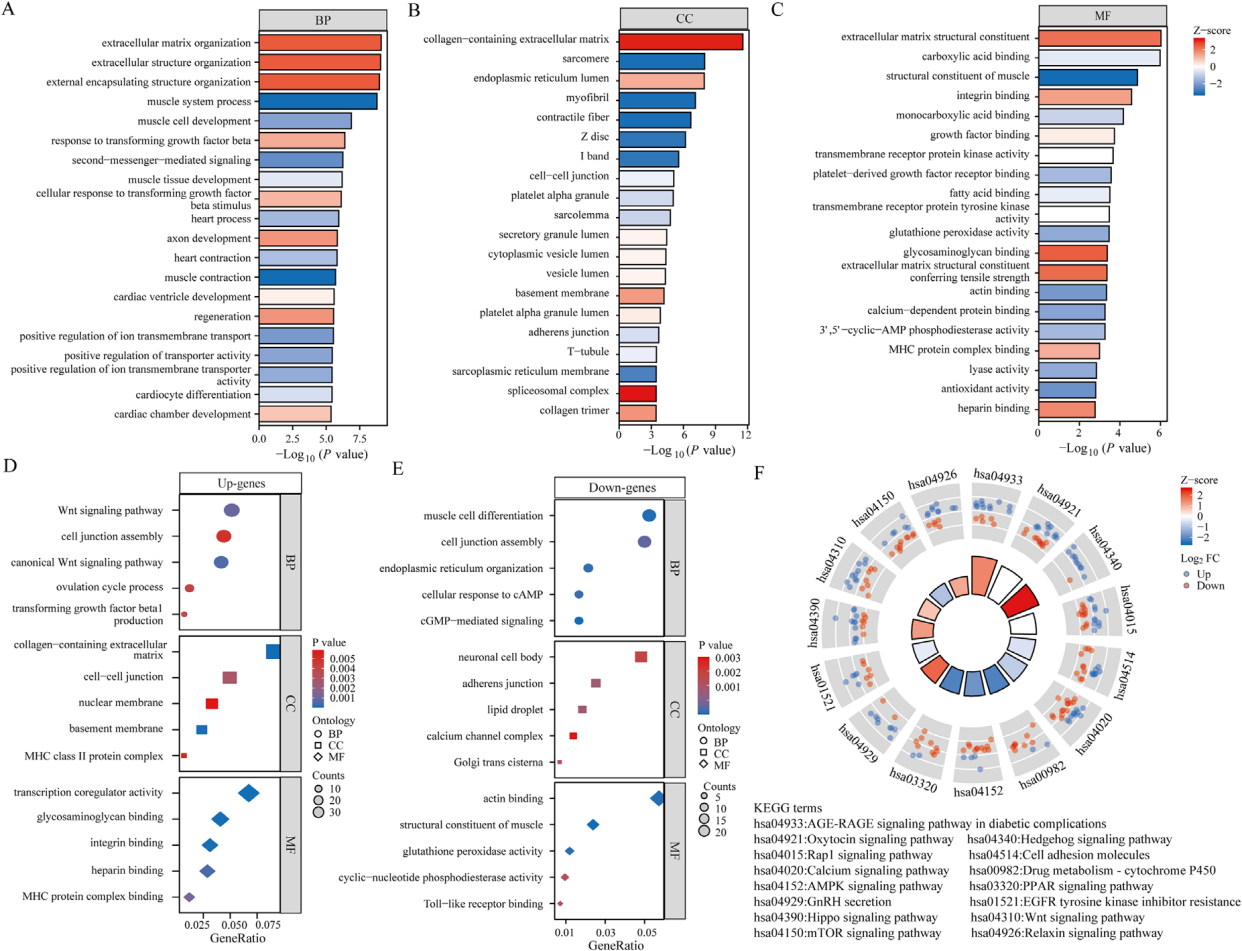
Functional analysis of BPH differential genes. **(A-C)** Bar graph showing the results of GO analysis of differential genes (including BP, CC and MF). **(D)** Bubble plots showing the results of GO analysis of up-regulated differential genes. **(E)** Bubble plots showing the results of GO analysis of down-regulated differential genes. **(F)** Results of KEGG analysis of differential genes.

In order to provide a deeper understanding of the roles played by BPH-associated genes, an analysis was performed on the functional enrichment using GO. This tool is commonly used for the annotation of gene functions across MF, BP, and CC categories. In the BP module, 185 genes were identified to be linked with pathways related to the extracellular matrix organization, extracellular structure organization, external encapsulating structure organization, and regulation of cellular response to transforming growth factor beta stimulus (**Figure 2A**). Analysis within the CC module revealed that these 185 genes were linked to collagen-containing extracellular matrix, sarcomere, endoplasmic reticulum lumen, and myofibril (**Figure 2B**). Furthermore, examination of the MF module indicated that these genes were associated with extracellular matrix structural constituent, carboxylic acid binding, muscle structural components, and integrin binding (**Figure 2C**). Subsequently, we performed GO analysis separately on 67 up-regulated genes and 118 down-regulated genes among the differential genes. Our findings showed that upregulated genes were connected to the Wnt signaling pathway, collagen-containing extracellular matrix, and transcriptional co-regulator activity (**Figure 2D**). Conversely, downregulated genes were found to be linked to muscle cell differentiation, neuronal cell bodies, and actin binding (**Figure 2E**). KEGG Enrichment Analysis is a valuable tool for investigating gene functions and high-level genome functional information. In our study, we performed KEGG analysis on 185 genes and identified several well-known pathways associated with differential genes in BPH. These pathways include the AGE-RAGE signaling pathway in diabetic complications, Oxytocin signaling pathway, Hedgehog signaling pathway, AMPK signaling pathway, and mTOR signaling pathway (**Figure 2F**).

### 3.3 Machine learning approach to screen BPH-related signature genes

**Figure 3:**
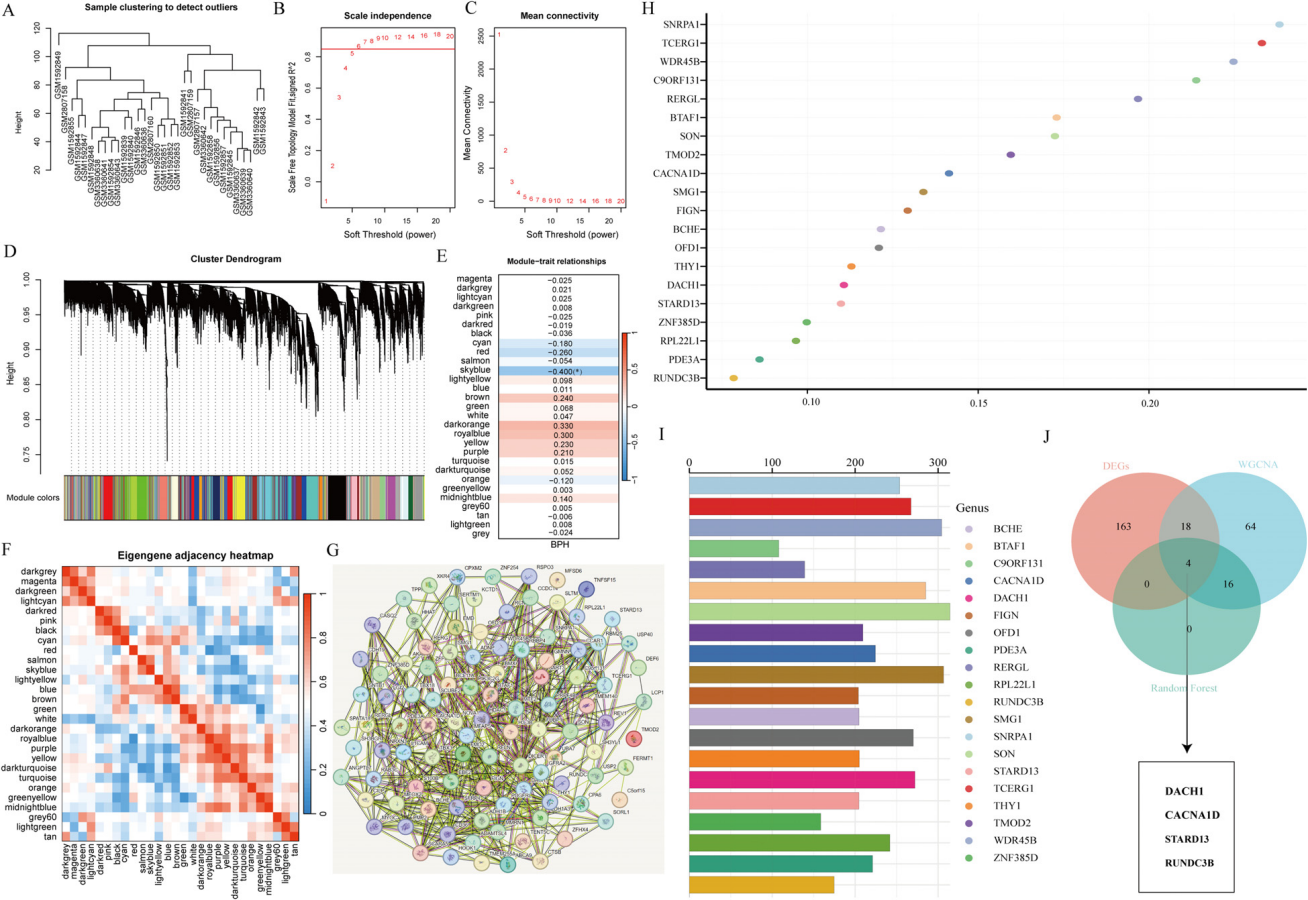
Screening characteristic genes related to BPH. **(A)** Sample clustering to detect outliers. **(B, C)** Determining soft thresholds using network topology analysis. **(D)** Construction of co-expression networks based on soft thresholds. **(E)** Relationship between sample features and modules. **(F)** Correlation analysis among different modules. **(G)** Interaction network diagram of genes in the skyblue module. **(H, I)** Randomforest algorithm identifies top 20 most BPH-associated genes. **(J)** Venn diagram showing the intersection of common feature genes.

To identify optimal biomarkers for BPH, our study conducted a thorough analysis using various machine learning techniques. By analyzing the expression profiles of BPH-related genes with default parameters in WGCNA (**Figures 3A–C**), we discovered 29 significant co-expression modules (**Figure 3D**). Our examination of module-trait correlations revealed a strong association between BPH-related genes in the azure module and BPH (**Figure 3E**). Subsequently, we focused on genes within the skyblue module and identified 103 relevant genes. We also investigated the relationships among the 29 modules (**Figure 3F**) and constructed an interaction network diagram of the 103 BPH-related genes in the skyblue module (**Figure 3G**). To enhance our understanding of BPH markers, we utilized the random forest algorithm to identify the top 20 markers from the 103 important genes identified by WGCNA (**Figures 3H, I**). Ultimately, our analysis pinpointed DACH1, CACNA1D, STARD13, and RUNDC3B as the most crucial biomarkers associated with BPH among BPH-related genes (**Figure 3J**).

### 3.4 Validation of DACH1, CACNA1D, STARD13 and RUNDC3B in BPH

**Figure 4:**
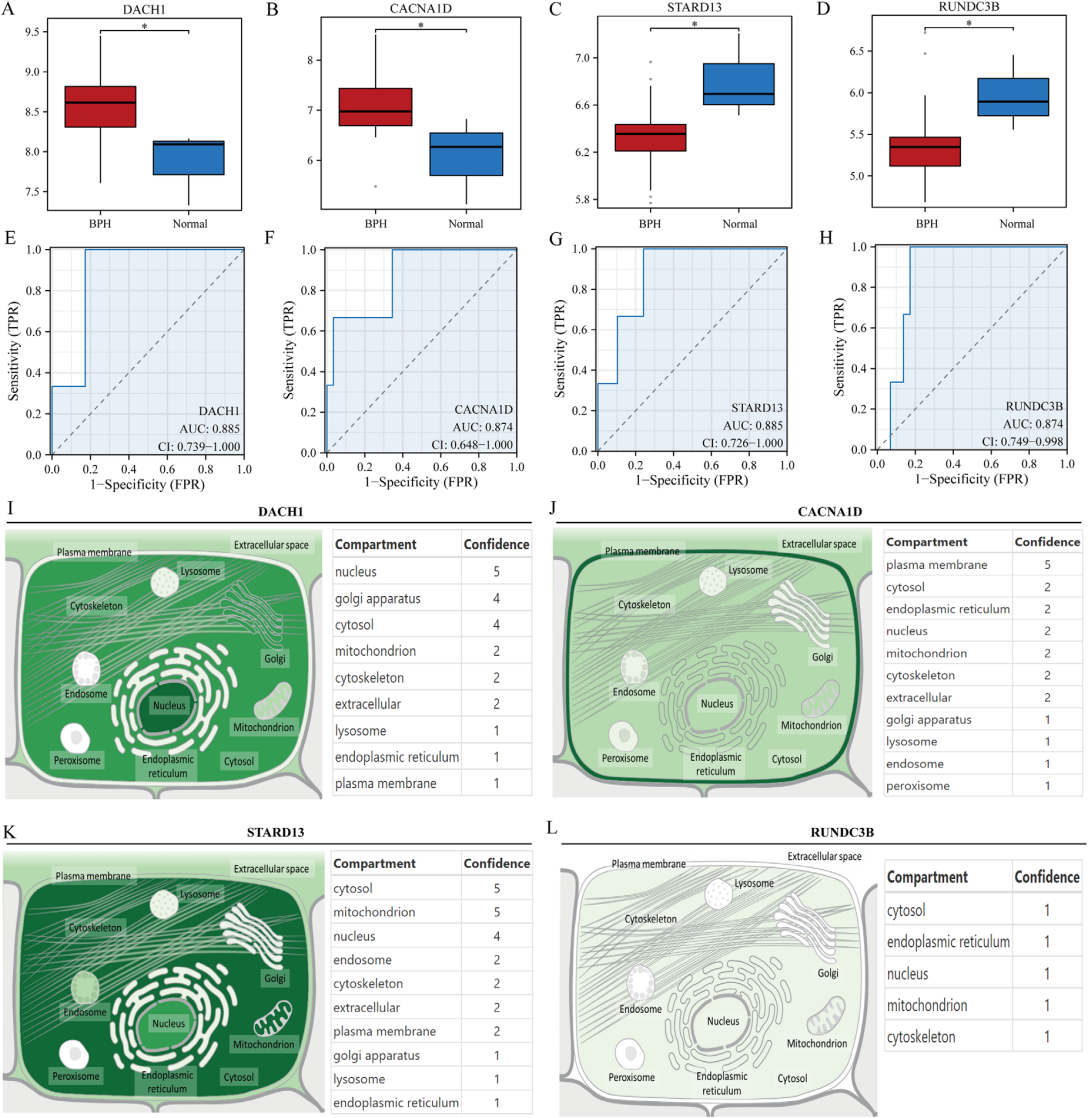
Validation of key genes. **(A-D)** Differential expression of DACH1, CACNA1D, STARD13 and RUNDC3B in hyperplastic and normal prostate samples. **(E-H)** Predictive value of DACH1, CACNA1D, STARD13 and RUNDC3B in the diagnosis of BPH. **(I-L)** Localization analysis of DACH1, CACNA1D, STARD13 and RUNDC3B in cells. *P<0.05.

Initially, the research examined the variation in expression of four genes (DACH1, CACNA1D, STARD13, and RUNDC3B) in samples of hyperplastic and normal prostates. Findings revealed that DACH1 and CACNA1D demonstrated considerable upregulation in hyperplastic prostate specimens when compared to normal samples (**Figures 4A, B**). In contrast, STARD13 and RUNDC3B displayed noteworthy upregulation in normal prostate samples compared to hyperplastic samples (**Figures 4C, D**). ROC curves were then used to assess the predictive potential of these key gene markers in prostate enlargement, revealing AUC values of 0.885, 0.874, 0.885, and 0.874 for DACH1, CACNA1D, STARD13 and RUNDC3B, respectively (**Figures 4E–H**). These findings suggest that these genes may serve as valuable predictors in diagnosing prostate enlargement. Subsequently, the subcellular localization of these four genes was analyzed using the Genecards website, showing that DACH1 was predominantly localized in the nucleus, CACNA1D in the plasma membrane, STARD13 in the cytosol, and there was no difference in the expression of RUNAC3B at different cellular locations (**Figures 4I–L**).

### 3.5 A network of regulatory mechanisms for DACH1, CACNA1D, STARD13 and RUNDC3B

**Figure 5:**
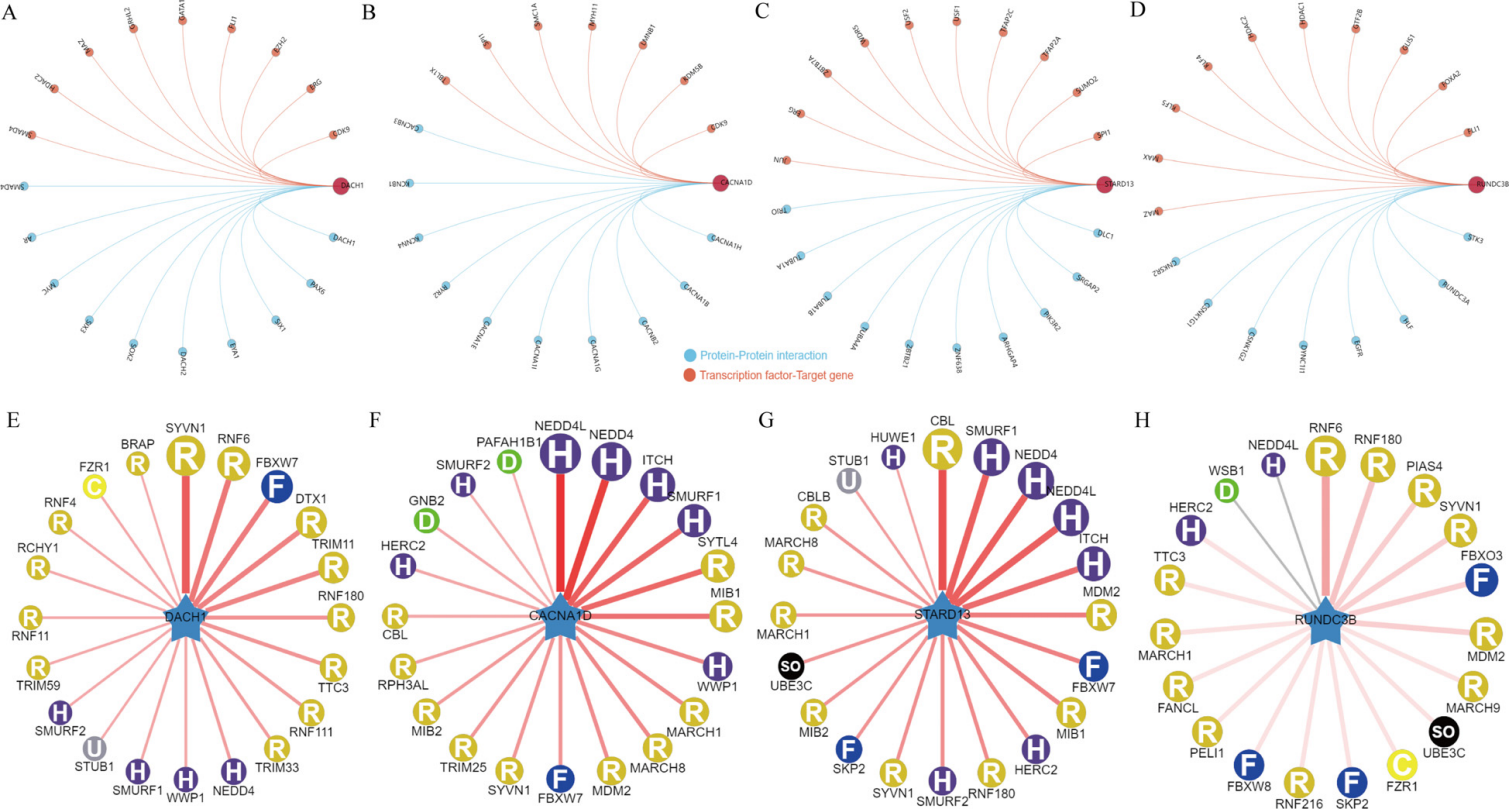
Regulatory network of key genes for BPH. **(A-D)** Interaction network map of DACH1, CACNA1D, STARD13 and RUNDC3B and regulatory network of transcription factors. **(E-H)** DACH1, CACNA1D, STARD13 and RUNDC3B-related E3 ubiquitin ligases.

In order to delve deeper into the potential regulatory mechanisms of genes associated with BPH, an in-depth analysis was carried out on the aforementioned four pivotal genes. Initially, we examined the interaction network of these genes and also investigated the transcription factor regulatory network (**Figures 5A–D**). A common transcriptional regulator, CDK9, was identified for DACH1 and CACNA1D. Additionally, a common transcriptional regulator, ERG, was found for DACH1 and STARD13, and a common transcriptional regulator, MAZ, was discovered for DACH1 and RUNDC3B, HDAC2, and FLI1. Furthermore, a common transcriptional regulator, SPI1, was recognized for CACNA1D and STARD13. In addition, we analyzed which E3 ubiquitin ligases are associated with DACH1, CACNA1D, STARD13 and RUNDC3B when used as substrates. We show the top 20 most relevant E3 ubiquitin ligases (**Figures 5E–H**). We found that the E3 ubiquitin ligase SYVN1 is correlated with DACH1, CACNA1D, STARD13 and RUNDC3B.

### 3.6 Functional analysis of key genes for BPH

**Figure 6:**
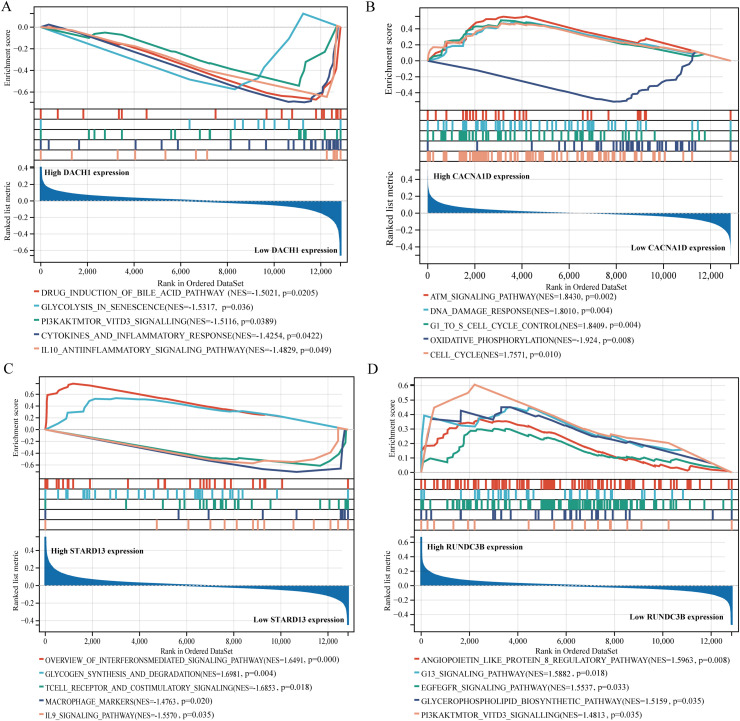
Gene enrichment analysis of key genes. **(A)** Grouped by high and low DACH1 expression and analyzed for gene enrichment. **(B)** Grouped by high and low CACNA1D expression and analyzed for gene enrichment. **(C)** Grouped by high and low STARD13 expression and analyzed for gene enrichment. **(D)** Grouped by high and low RUNDC3B expression and analyzed for gene enrichment.

To further investigate the potential mechanism of action of DACH1, CACNA1D, STARD13, and RUNDC3B in BPH, we conducted gene enrichment analysis on these genes. Our findings revealed that DACH1 was significantly associated with drug induction of the bile acid pathway, glycolysis in senescence, PI3K/AKT/MTOR/VITD3 signaling, cytokines and inflammatory response, and IL10 anti-inflammatory signaling pathway (**Figure 6A**). CANA1D is primarily associated with the ATM signaling pathway, DNA damage response, GI to S cell cycle control, oxidative phosphorylation, and cell cycle (**Figure 6B**). STARD13 is mainly related to the overview of interferons-mediated signaling pathway, glycogen synthesis and degradation, T cell receptor and costimulatory signaling, macrophage markers, and IL9 signaling pathway (**Figure 6C**). RUNDC3B is mainly related to the angiopoietin-like protein 8 regulatory pathway, G13 signaling pathway, EGF/EGFR signaling pathway, glycerophospholipid biosynthetic pathway, and PI3K/AKT/MTOR/VITD3 signaling (**Figure 6D**).

### 3.7 Analysis of immune cell infiltration of key genes in BPH samples

**Figure 7:**
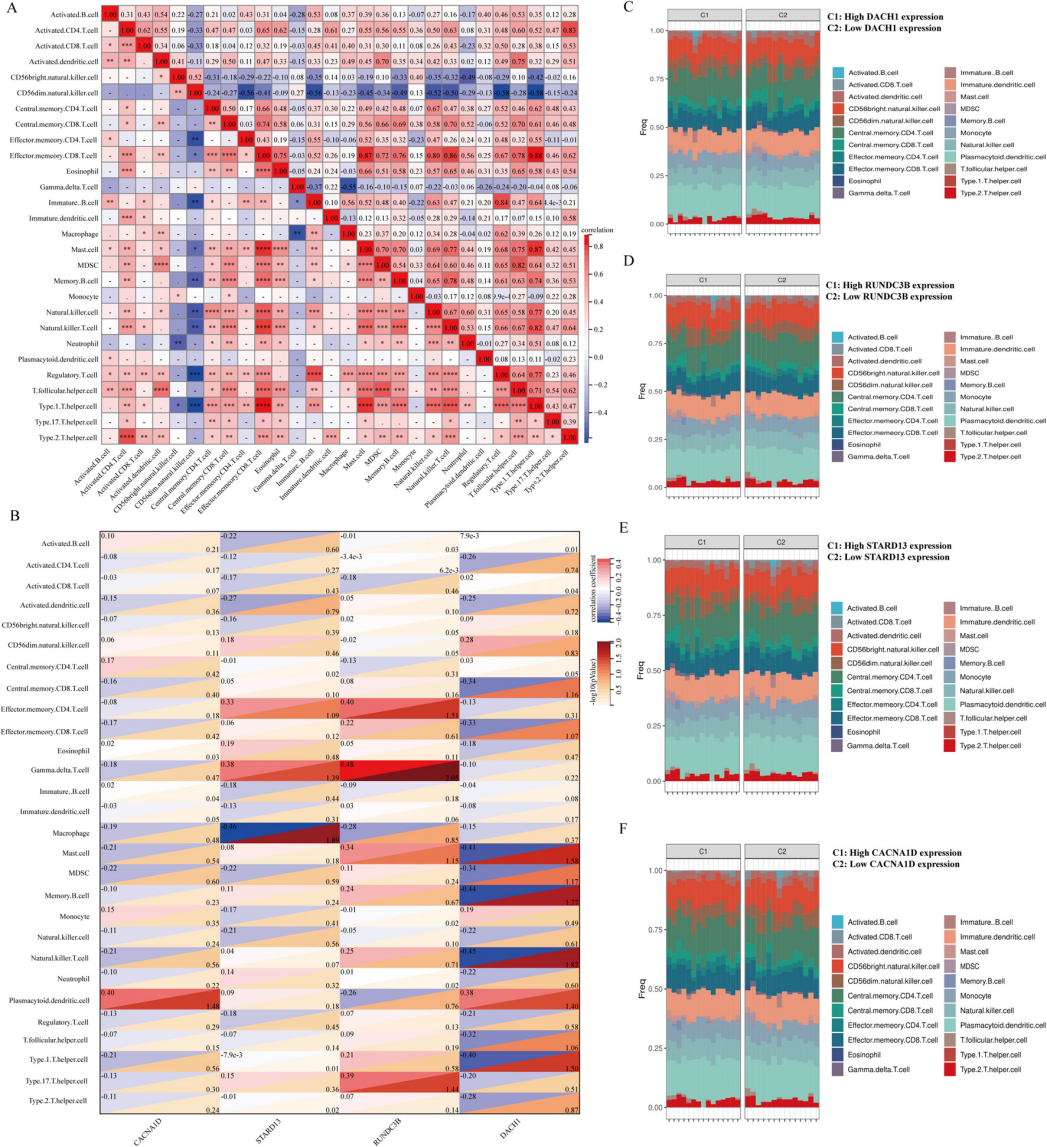
Relationship between key characterized genes and immune cells in BPH samples. **(A)** Heatmap of the correlation of 28 immune cells. **(B)** Heatmap of DACH1, CACNA1D, STARD13, and RUNDC3B correlation with 28 immune cells with correlation coefficients in the upper left and p-values in the lower right. **(C-F)** Percentage abundance of infiltrating immune cells per sample after grouping based on characterized genes.

There is a growing body of experimental and clinical evidence indicating that immune mechanisms may hasten the advancement of BPH. This has led us to investigate the connection between key characteristics and immune infiltration in BPH. In their study, Pornpimol et al. identified 28 genes that are indicative of immune cells, and we utilized the ssGSEA method to estimate patient immune cell scores based on this set of genes. A heat map was created to display the connection between the levels of infiltration of 28 distinct immune cells. The examination indicated an inverse relationship between CD56bright natural killer cells and CD56dim natural killer cells with other immune cells, whereas the levels of infiltration of the remaining immune cells demonstrated predominantly positive connections (**Figure 7A**). We then analyzed the correlation between DACH1, CACNA1D, STARD13 and RUNDC3B with these 28 immune cells and plotted heat maps. The study revealed that CACNA1D expression was significantly associated with the infiltration levels of Eosinophil and Immature B cells. Additionally, STARD13 expression was found to be mainly related to the infiltration levels of Central memory CD4 T cells, Type 1 T helper cells, and Type 2 T helper cells. Furthermore, the expression of RUNDC3B was significantly correlated with the infiltration levels of Activated B cells, Activated CD4 T cells, Monocytes, and Neutrophils. Lastly, DACH1 expression was notably correlated with the infiltration levels of Activated B cells and Activated CD8 T cells (**Figure 7B**). The BPH samples were grouped based on the expression levels of DACH1, CACNA1D, STARD13, and RUNDC3B. The abundance of immune cells in each sample was visualized using a heatmap, with samples grouped by high and low expression levels. Different colors were used to represent various immune cell types (**Figures 7C–F**).

### 3.8 Correlation of key characterization genes with therapeutic drugs for BPH treatment

**Figure 8:**
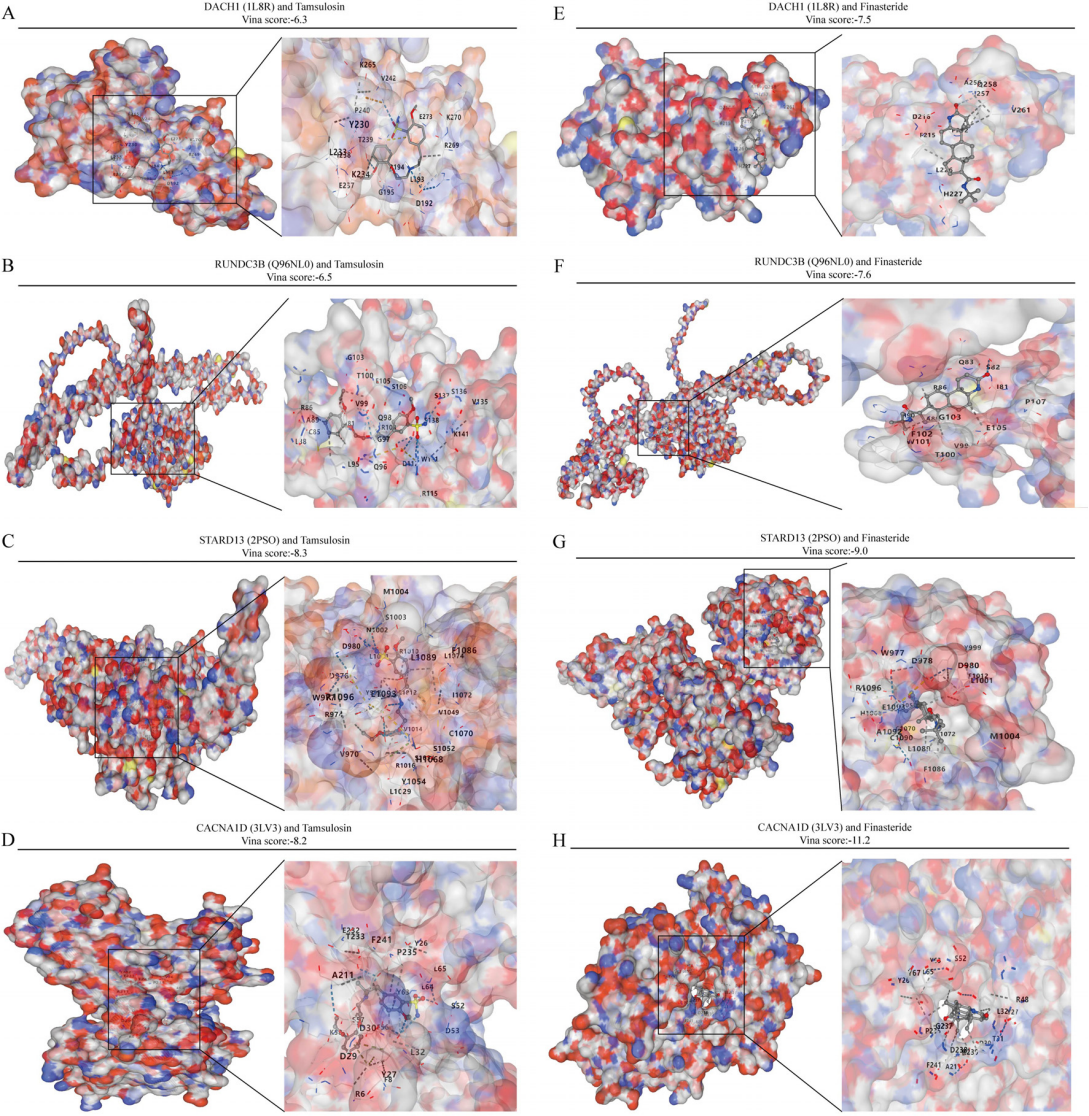
Analysis of the binding capacity of characterized genes to tamsulosin and finasteride. **(A-D)** Molecular docking of characterized genes with tamsulosin. **(E-H)** Molecular docking of characterized genes with finasteride.

Commonly used medications for prostate enlargement include 5-alpha reductase inhibitors and alpha receptor blockers. Tamsulosin represents alpha-blockers, while finasteride represents 5-alpha reductase inhibitors. In this study, we conducted molecular docking of these two drugs with DACH1, CACNA1D, STARD13, and RUNDC3B to assess their interaction. The vina scores for DACH1 after docking with tamsulosin and finasteride were -6.3 and -7.5, respectively. For RUNDC3B, the vina scores after docking with tamsulosin and finasteride were -6.5 and -7.6. STARD13 showed vina scores of -8.3 and -9.0 after docking with tamsulosin and finasteride. Finally, the vina scores for CACNA1D after docking with tamsulosin and finasteride were -8.2 and -11.2 (**Figures 8A–H**). These results indicate that the four characterized genes, DACH1, CACNA1D, STARD13 and RUNDC3B, have better binding activities with tamsulosin and finasteride.

### 3.9 Expression verification of target genes

**Figure 9:**
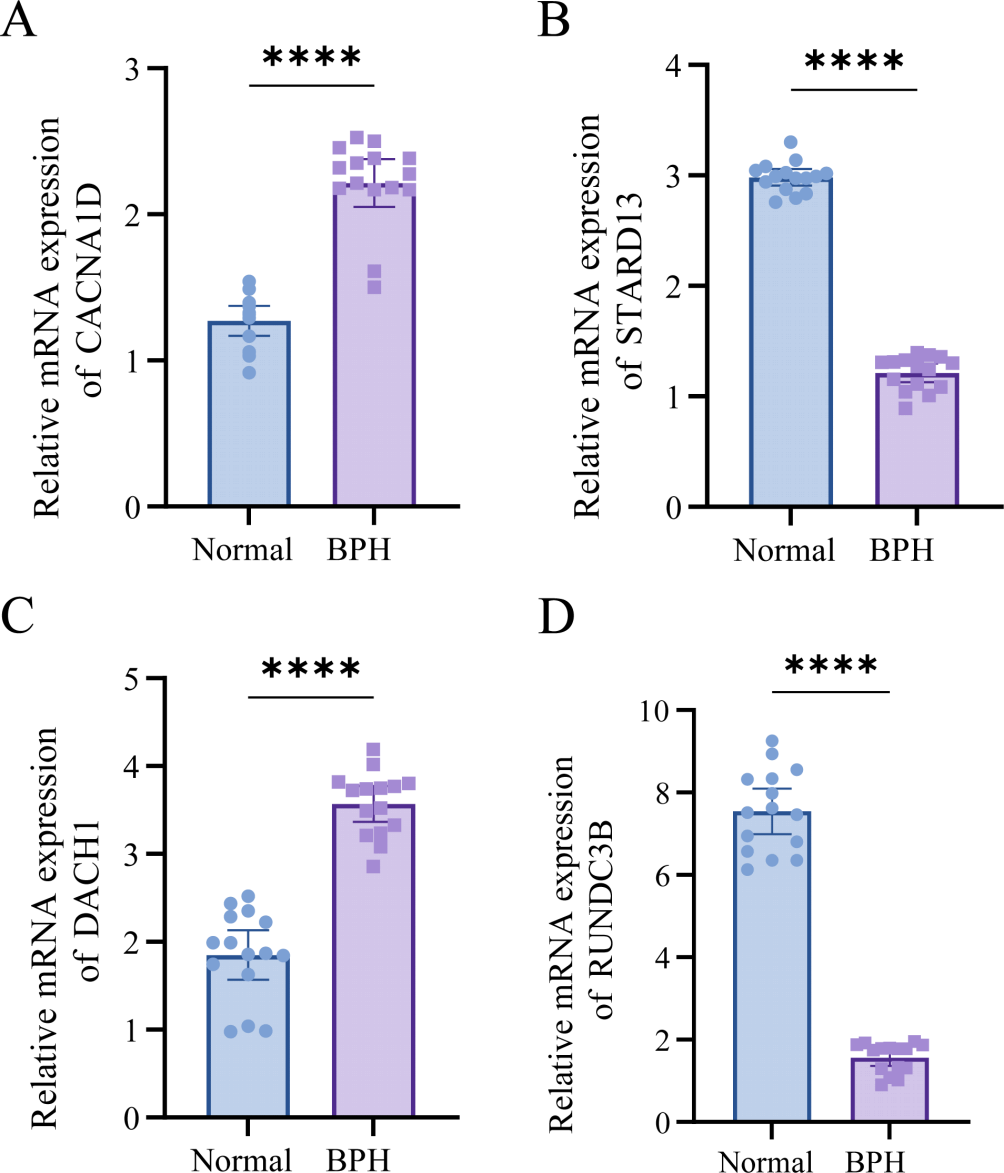
DACH1, CACNA1D, STARD13, and RUNDC3B are highly expressed in BPH tissues. **(A-D)** Differential expression of DACH1, CACNA1D, STARD13, and RUNDC3B in BPH and normal tissues. ****P <0.0001.

Based on our analyses, we identified the significant roles of DACH1, CACNA1D, STARD13, and RUNDC3B in BPH. For further investigation, we collected five cases of BPH tissue alongside normal prostate tissue. Our analysis revealed that the expression levels of DACH1 and CACNA1D in BPH tissues were significantly higher than those in normal prostate tissues, whereas the expression levels of STARD13 and RUNDC3B were significantly lower in BPH tissues compared to normal prostate tissues (**Figures 9A–D**, **Table 1**). These findings are consistent with our previous analyses and suggest that these markers may serve as novel diagnostic indicators for BPH.

**Table 1:** The primer sequence for the target gene.

Genes	Forward primer sequence (5’-3’)	Reverse primer sequence (5’-3’)
DACH1	GAGCGAGACCCACACAACAA	AGTGTCCCGCAAGTCGAAAT
CACNA1D	AAAGAGAGAGCTTGGGTGGC	GGTCCCTGCCCTTATTCTCG
STARD13	GCCGAGATGTTCAGTCAGGT	CACTAGCTGATGGCGTGCTA
RUNDC3B	AACTGCCCATCTCACCAACC	ACCACTGTTTATTGTGTTTTCGG
GAPDH	GTATTGCTGACCGTATGC	CTGCCTCATCGTATTCCT

## 4 Discussion

Identifying distinct indicators and clarifying their fundamental mechanisms in BPH is essential for improving predictive diagnosis, targeted prevention, and personalized treatment of the condition. Our research indicates that DACH1, CACNA1D, STARD13, and RUNDC3B are critical genes linked to BPH, demonstrating robust diagnostic potential. This investigation introduces fresh data proposing that DACH1, CACNA1D, STARD13, and RUNDC3B may function as compelling diagnostic indicators for BPH. The development of multi-omics technology has significantly enriched our comprehension of the molecular basis of BPH and facilitated ongoing progress in clinical diagnostic and therapeutic strategies for BPH.

In this study, we utilized three BPH-related datasets: GSE65343, GSE104749, and GSE119195. Differential analysis revealed 185 genes, with 67 up-regulated and 118 down-regulated. GO analysis identified pathways involving extracellular matrix organization and cell response to transforming growth factor beta stimulation. KEGG analysis found that 185 differential genes were related to many well-known signaling pathways such as AMPK signaling pathway and WNT signaling pathway. Recent studies have demonstrated that the flower of Hosta plantaginea (Lam.) Aschers modulates inflammation and amino acid metabolism by inhibiting the NF-κB, MAPK, JAK-STAT, PI3K-Akt, and AMPK pathways, thereby alleviating BPH in rats (22). Furthermore, these studies underscore the significant role of the MAPK signaling pathway in BPH (23). Additionally, Di-(2-ethylhexyl) phthalate has been shown to promote benign prostatic hyperplasia through the KIF11-Wnt/β-catenin signaling pathway (24). Moreover, MicroRNA-340 inhibits epithelial-mesenchymal transition by impairing the ROCK-1-dependent Wnt/β-catenin signaling pathway in epithelial cells derived from human benign prostatic hyperplasia. Collectively, these findings highlight the crucial involvement of the WNTK signaling pathway in BPH (25). BPH, as a disease associated with aging, advances characterized by ongoing inflammation and remodeling of the ECM surrounding BPH nodules (26). The differential genes we analyzed were also confirmed to be related to ECM, further supporting the idea that the differential genes we identified are closely associated with BPH. In addition, we also investigated the potential regulatory mechanisms of up-regulated and down-regulated genes separately. Our results suggested that these genes are significantly connected with MHC class II protein complexes as well as Toll-like receptors. It is widely recognized that the presentation of antigens via MHC class II molecules is crucial for triggering effective immune responses mediated by CD4 T cells and for sustaining tolerance toward self-antigens (27). Toll-like receptors, members of the pattern recognition receptor family, are predominantly expressed by innate immune cells like dendritic cells, macrophages, monocytes, neutrophils, and epithelial cells, acting as the first line of defense in the human body (28). Therefore, we hypothesize that differential genes linked to BPH may play a significant role in immune regulation through MHC class II protein complexes and Toll-like receptors.

Two machine learning strategies, WGCNA and RF, were utilized to identify key diagnostic markers for BPH. By intersecting markers from both algorithms, the number of markers was reduced to enhance specificity and sensitivity. Ultimately, DACH1, CACNA1D, STARD13 and RUNDC3B were identified as key diagnostic markers for BPH. Subsequently, we analyzed the potential mechanisms through which these four key genes operate. We conducted a mapping of the interaction network involving these four key genes and the transcription factor regulatory network. DACH1, CACNA1D, STARD13 and RUNDC3B are implicated in potentially regulating the progression of BPH through these genes. Protein ubiquitination plays a crucial role in controlling protein abundance and function. It is implicated in diverse cellular processes such as protein degradation, DNA repair, gene expression, and signal transduction (29, 30). Dysregulation of this intricate system is closely linked to a range of human diseases, including prostatic hyperplasia. Therefore, we also analyzed the top 20 most relevant E3 ubiquitin ligases when DACH1, CACNA1D, STARD13 and RUNDC3B were used as substrates.

To delve deeper into the potential mechanisms of DACH1, CACNA1D, STARD13, and RUNDC3B in regulating prostate hyperplasia, we stratified prostate hyperplasia samples according to the levels of expression of these genes and conducted GSEA. Our research revealed that DACH1 is primarily linked to cytokines and inflammatory responses in prostatic hyperplasia samples. There is increasing evidence suggesting that inflammation-induced tissue damage and wound healing can trigger uncontrolled proliferation of epithelial and stromal cells. Tissue fibrosis caused by inflammation is characterized by active epithelial-mesenchymal transition, myofibroblast differentiation, excessive ECM deposition, and heightened mechanical stiffness (31). Prostatic fibrosis plays a role in ECM remodeling and negatively impacts tissue elasticity and compliance, thereby exacerbating LUTS in patients with BPH (32). CACNA1D is primarily related with the cell cycle, potentially impacting the development of prostatic hyperplasia cells through cell cycle regulation. In the progression of BPH, uncontrolled cell proliferation and decreased apoptosis contribute to an overall increase in prostate cell numbers (33). The cell cycle governs the division and replication of prostate cells, consisting of four stages. If the G1/S checkpoint is disrupted, unchecked DNA replication occurs, causing abnormal cell proliferation (34). Furthermore, our findings suggest a correlation between CACNA1D and the G1/S checkpoint, indicating a potential mechanism by which it regulates prostate hyperplasia. STARD13 has been implicated in macrophage activity. Recent studies show that macrophages have a role in immune inflammation and proliferation in benign prostatic hyperplasia through the androgen receptor and CD40/CD40L signaling pathway (35). This pathway may also be crucial for the functioning of STARD13. It was also discovered that RUNDC3B has a close association with angiogenesis. Despite being a benign condition, BPH arises from uncontrolled and excessive growth in stromal and epithelial regions (36). Consequently, areas of rapid growth in BPH become hypoxic, leading to the induction of angiogenesis (37). This suggests a potential mechanism through which RUNDC3B may influence the progression of prostatic hyperplasia by modulating angiopoietin like protein 8.

In light of the pivotal role of immunity in BPH, our study delved into the interplay between key diagnostic genes and immune cells in BPH. Our findings suggest a significant correlation between CACNA1D expression and the presence of Eosinophils and Immature B cells. Additionally, STARD13 expression appears to be strongly associated with the infiltration of Central memory CD4 T cells, Type 1 T helper cells, and Type 2 T helper cells. RUNDC3B expression is notably linked to higher levels of Activated B cells, Activated CD4 T cells, Monocytes, and Neutrophils. Furthermore, DACH1 expression shows a notable correlation with increased infiltration of Activated B cells and Activated CD8 T cells. These data provide new insights into the development and immune mechanisms of DACH1, CACNA1D, STARD13 and RUNDC3B during BPH. Tamsulosin and finasteride are frequently prescribed medications for treating BPH and are integral to its clinical management (38, 39). Our study utilized molecular docking techniques to establish a strong correlation between DACH1, CACNA1D, STARD13 and RUNDC3B and these drugs, suggesting that these genes may serve as promising drug targets for BPH. This study suggests that the genes DACH1, CACNA1D, STARD13, and RUNDC3B could play a significant role in the development of BPH and could potentially be targeted for BPH treatment. Further research on these genes and pathways is crucial to validate the findings of this study.

Our research primarily relies on online datasets for analysis, which inherently presents certain limitations. In future studies, we will concentrate on investigating the functions of DACH1, CACNA1D, STARD13, and RUNDC3B, as well as further validating these biomarkers in clinical samples. We aim to explore the relationship between their expression levels and the progression of BPH to elucidate their roles at different stages of the disease. Additionally, we will examine how these biomarkers affect the prostatic hyperplasia process through specific cell signaling pathways and immune responses, particularly their involvement in chronic inflammation and cell proliferation.

## 5 Conclusion

This study utilized a combination of bioinformatics techniques and machine learning algorithms to investigate the functional enrichment and pathways of differentially expressed genes in BPH. The identified genes DACH1, CACNA1D, STARD13, and RUNDC3B are suggested to be key players in the pathogenesis of BPH, providing valuable insights for a deeper comprehension of this condition.

## Data Availability

The original contributions presented in the study are included in the article/supplementary material. Further inquiries can be directed to the corresponding authors.
